# Application of the iPLUS non-coding sequence in improving biopharmaceuticals production

**DOI:** 10.3389/fbioe.2024.1355957

**Published:** 2024-02-06

**Authors:** Inês Reis-Claro, Maria Inês Silva, Ana Moutinho, Beatriz C. Garcia, Isabel Pereira-Castro, Alexandra Moreira

**Affiliations:** ^1^ Gene Regulation, i3S—Instituto de Investigação e Inovação em Saúde, Universidade do Porto, Porto, Portugal; ^2^ IBMC—Instituto de Biologia Molecular e Celular, Universidade do Porto, Porto, Portugal; ^3^ ICBAS—Instituto de Ciências Biomédicas Abel Salazar, Universidade do Porto, Porto, Portugal

**Keywords:** *cis*-regulatory sequences, mRNA therapeutics, recombinant protein expression, USE, 3′UTR, iPLUS

## Abstract

The biotechnological landscape has witnessed significant growth in biological therapeutics particularly in the field of recombinant protein production. Here we investigate the function of 3′UTR *cis*-regulatory elements in increasing mRNA and protein levels in different biological therapeutics and model systems, spanning from monoclonal antibodies to mRNA vaccines. We explore the regulatory function of iPLUS - a universal sequence capable of consistently augmenting recombinant protein levels. By incorporating iPLUS in a vector to express a monoclonal antibody used in immunotherapy, in a mammalian cell line used by the industry (ExpiCHO), trastuzumab production increases by 2-fold. As yeast *Pichia pastoris* is widely used in the manufacture of industrial enzymes and pharmaceuticals, we then used iPLUS in tandem (3x) and iPLUSv2 (a variant of iPLUS) to provide proof-of-concept data that it increases the production of a reporter protein more than 100-fold. As iPLUS functions by also increasing mRNA levels, we hypothesize that these sequences could be used as an asset in the mRNA vaccine industry. In fact, by including iPLUSv2 downstream of Spike we were able to double its production. Moreover, the same effect was observed when we introduced iPLUSv2 downstream of MAGEC2, a tumor-specific antigen tested for cancer mRNA vaccines. Taken together, our study provides data (TLR4) showing that iPLUS may be used as a valuable asset in a variety of systems used by the biotech and biopharmaceutical industry. Our results underscore the critical role of non-coding sequences in controlling gene expression, offering a promising avenue to accelerate, enhance, and cost-effectively optimize biopharmaceutical production processes.

## 1 Introduction

The recombinant protein production market has grown immensely over the past years, with a strong impact in biological therapeutics (O’Flaherty et al., 2020; Tihanyi and Nyitray, 2020). Since the first time the Food and Drug Administration (FDA) approved insulin to be used as a therapeutics, 41 years have passed and several hundred more have been approved (Dingermann, 2008; Kesik-Brodacka, 2018). Recombinant proteins of therapeutic interest include monoclonal antibodies, hormones, enzymes, among other classes of proteins and/or peptides (O’Flaherty et al., 2020). Other biopharmaceutical whose importance and usage rose with the COVID-19 pandemic is the mRNA vaccines. mRNA as a therapeutics has the advantage of delivering the specific sequence of a known antigen, instructing the cells to manufacture the protein, which then culminates in an immunologic response specific for that immunogen (Pardi et al., 2018). Additionally, as they are simple to use and manufacture, mRNA vaccines have a variety of applications, from therapeutic vaccination for multiple cancers, to preventive immunization towards different pathogens, including new pandemics and outbreaks (Rauch et al., 2018).

The mRNAs untranslated regions (UTRs) have been known for a long time to have a crucial role in gene expression regulation (Gruber and Zavolan, 2019; Pereira-Castro and Moreira, 2021). There is a large variety of *cis*-regulatory elements present in the UTRs, either in the 5′ or the 3′ (Matoulkova et al., 2012). The 5′UTR has an important role in translation initiation, becoming an important region for regulation of translational efficiency (Jackson et al., 2010). For example, the presence of the internal ribosomal entry sites (IRES) sequence in the 5′UTR, found originally in certain virus, promotes the recruitment of the ribosomes, improving translation initiation. As so, this sequence is commonly employed in different expression systems (Johnson et al., 2017). The Kozak motif is also a commonly employed 9-nucleotide conserved sequence in expression vectors, as it is critical for the recognition of the translation initiation site by the ribosomes in eukaryotic mRNAs (Hernández et al., 2019). As for the 3′UTRs, their ability to control the mRNA fate comes from numerous *cis*-regulatory elements, by allowing the binding of *trans*-acting factors, such as RNA-binding proteins (RBPs) and microRNAs (miRNAs) (Mayr, 2019). The binding of these factors will impact gene expression regulation, not only by affecting the protein expression levels, but also other important factors for the fate of the transcript, namely, its stability, subcellular localization, translation efficiency and the function of the resulting protein (Mayr, 2017; Pereira-Castro and Moreira, 2021). Usually, each 3′UTR regulatory sequence is tested in a gene and cell-type specific context (Vallazza et al., 2015). Sequences from the α-globin and β-globin, for example, are highly employed in expression systems since it is known that, in a physiological context, they promote a high stability to their mRNAs (Jiang et al., 2006). Another example of a broadly used sequence, is the Woodchuck Hepatitis Virus Posttranscriptional Regulatory Element (WPRE), a 597 nucleotide-long sequence, that has been showed to increase luciferase and GFP production levels by five to eight-fold, when subcloned in the 3′ of the cDNAs subcloned in expression vectors (Zufferey et al., 1999).

Recently, genome-wide studies have been conducted in the search for active regulatory sequences naturally present in the genomes, reveling new sequences that may be employed in the production of recombinant proteins of high commercial value by the biopharma and biotech industries (Sample et al., 2019; Cao et al., 2021; Leppek et al., 2022; Kirshina et al., 2023). For the 5′UTR, Cao et al. (2021) employed high-throughput screening in combination with sequence engineering, by initially analyzing 5′UTRs that naturally reveal good translation efficiencies, followed by synthetic modification of these sequences to improve their effectiveness even further. The results obtained were validated in several cell models and the increase of protein expression was corroborated also in proteins with therapeutic value, such as vascular endothelial growth factor (VEGF). This approach has also been applied to the search for regulatory regions in the 3′UTR, also in the context of mRNA-based therapeutics, such as cancer vaccines, by increasing the mRNA stability and protein expression (Leppek et al., 2022; Kirshina et al., 2023). For example, the application of a combination of mtRNR1 (mitochondrial rRNA) and AES (Amino-Terminal Enhancer Of Split) 3′UTR sequences, promoted a T cell immune response against a vaccine antigen, as well as an increase in the delivery of reprogramming factors to induce pluripotency of differentiated cells (Orlandini von Niessen et al., 2019). These efforts to find new non-coding regulatory sequences reveal a yet unexplored field to optimize the expression vectors used in the production of biological therapeutics.

Certainly, *cis*-regulatory sequences present in the UTRs can be used to increase mRNA expression, as well as protein production, either in large scale as for recombinant protein production, or in the cells in the context of an immunization with an mRNA vaccine (Gómez-Aguado et al., 2020). For example, the human albumin 3′UTR increases the production of secreted luciferase by 2-3 fold in CHO cells (Pearson et al., 2012). The structure of the mRNAs in the vaccines mimics endogenous mRNAs (Pardi et al., 2018) and their design has been optimized to elicit a strong and specific immunological response. Delivery of the mRNA vaccines must promote the antigen’s expression at levels sufficient for detection of immune responses (Karikó et al., 1999). Thus, 5′UTR and 3′UTR secondary structures, 3′UTR length, and different *cis*-regulatory elements present in both the 3′UTR and 5′UTR contribute to achieve high expression levels (Gómez-Aguado et al., 2020; Jackson et al., 2020; Castillo-Hair and Seelig, 2022).

The upstream sequence elements (USEs), located upstream of polyadenylation signals (PAS), are a good example of *cis*-regulatory sequences present in the 3′UTR. USEs are involved in cleavage and polyadenylation (CPA) efficiency, by allowing the binding of the CPA machinery (Zhao et al., 1999; Proudfoot, 2011; Darmon and Lutz, 2012; Oliveira et al., 2019). USEs possess a characteristic pyrimidine and/or U-rich stretch (Chan et al., 2011) and function as binding sites for various RBPs, modulating gene expression (Carswell and Alwine, 1989; Moreira et al., 1998; Danckwardt et al., 2007). Therefore, USEs have the potential to be employed in different applications besides their physiological role in CPA. For example, the subcloning of different USEs in viral vectors was tested to improve their 3′end processing efficiency by avoiding readthrough, consequently increasing both their efficiency and biosafety (Schambach et al., 2007). Also, it was demonstrated that the use of the USE SV40-derived sequence, as well as its repetition in tandem 2 times, led to an increase of 45%–100% in gene expression, depending on the promoters used (Schambach et al., 2007).

We have previously found that *Drosophila*’s *polo* gene possesses a USE upstream of a weak PAS, highly conserved among *Drosophila* genomes (Oliveira et al., 2019). This 28-nucleotides long, pyrimidine-rich USE presents a physiological role in increasing Polo’s protein levels (Oliveira et al., 2019). This result led us to hypothesize that this mechanism is conserved, therefore, its function was investigated in proof-of-concept experiments in other genes and organisms. In *Danio rerio*, the microinjection of a plasmid containing this USE downstream of the green fluorescent protein (GFP) was used as a reporter and showed that the USE increases GFP expression levels by ∼2-fold, indicating that the function of the USE is conserved in vertebrates (Eufrásio et al., 2023). We found that three RBPs (HuR, hnRNP C and PTBP1) bind to the USE in human cells, and in *Drosophila,* Hephaestus (the ortholog of the human PTBP1), enhances the use of the PAS through interaction with *polo*´s USE leading to higher mRNA and protein levels (Oliveira et al., 2019; Eufrásio et al., 2023). In mammalian cell lines, specifically HeLa cells, it has previously been shown that this sequence led to an increase in a reporter’s activity (Eufrásio et al., 2023).

The consistent increase in protein levels observed when *polo*’s USE was inserted in the 3′UTR led us to secure its potential in biopharmaceutical’s formulations usage, patenting it as iPLUS, which stands for increase Protein Levels Universal Sequence (Oliveira et al., 2020). iPLUS fits in the need for non-coding regulatory regions to improve effectiveness of gene expression, as the biopharmaceutical and biotechnology industries requires their improvement in the manufacture processes with the goal of lowering production costs.

In this work we explored the potentialities of iPLUS in increasing the efficiency of mRNA and protein production for different biopharmaceuticals. We demonstrated that iPLUS, and a variant of this sequence that we named iPLUSv2, which consists of 3 repeats of the most conserved 8 nucleotide sequence stretch of iPLUS/USE (Oliveira et al., 2019) increases mRNA and protein levels in different models and for various proteins of biopharmaceutical interest. These results reinforce the importance of 3′ non-coding sequences in the regulation of gene expression, highlighting their potential use in the process of manufacture to accelerate, improve and reduce the costs of the biopharmaceutical’s production.

## 2 Materials and methods

### 2.1 Plasmids construction

#### 2.1.1 Trastuzumab_LC and HC

The plasmid pcDNA3.4-TOPO (Thermo Fisher Scientific) was used as a backbone to subclone the trastuzumab *LC* (light chain) and *HC* (heavy chain) upstream of the WPRE sequence. The pcDNA3.4-TOPO was digested with *Age*I-HF (NEB), the restriction site was blunted using Klenow (NEB), followed by digestion with *Xba*I (NEB). *LC* and *HC* were amplified by PCR using the phosphorylated primer pair LC+XbaI F; LC-R and HC+XbaI F; HC-R (Supplementary Table S1), pVITRO1-Trastuzumab-IgG1/κ [a gift from Andrew Beavil, Addgene plasmid #61883 (Dodev et al., 2014)] as the template DNA, and Phusion High Fidelity DNA polymerase (ThermoFisher Scientific) following manufacturer recommendations, for cloning in pcDNA3.4-TOPO in the *Xba*I and blunted *Age*I restriction sites.

#### 2.1.2 Trastuzumab_LC_iPLUS

Firstly, pGEM-4 (Promega) digested with *Sac*I (NEB) and *Kpn*I (NEB) was used to subclone the annealead oligos containing iPLUS sequence (AATTTATTTGTTTTTGCCCCTTCCCCTT—Supplementary Table S1) flanked by digested *Sac*I and *Kpn*I restriction sites (pGEM-4-iPLUS). pGEM-4-iPLUS was digested with *BamH*I (NEB) and *EcoR*I (NEB) in order to obtain the iPLUS sequence. Trastuzumab_LC was linearized by an inverted PCR using WPRE+BamHI-F and WPRE+EcoRI-R primers (Supplementary Table S1) and Phusion High Fidelity DNA polymerase (Thermo Fisher Scientific). Linearized plasmid was digested with *BamH*I and *EcoR*I to clone the iPLUS sequence downstream of the WPRE sequence and upstream the HSV TK PAS.

#### 2.1.3 Trastuzumab_HC_iPLUS

An inverted PCR using Trastuzumab_HC was performed as for Trastuzumab_LC_iPLUS. To obtain Trastuzumab_HC_iPLUS, the linearized plasmid and pGEM-4-iPLUS were digested with *Kpn*I and the restriction site was blunted using Klenow, followed by *EcoR*I digestion.

#### 2.1.4 pTZ-p04_GFP_iPLUS 3X and pTZ-p04_GFP_iPLUSv2

The pTZ-p04_GFP was already prepared and is a proprietary vector of Trenzyme GmbH. The iPLUS 3X (iPLUS in tandem three times) and iPLUSv2 (TTG​TTT​TTT​TGT​TTT​TTT​GTT​TTT) inserts were made by Trenzyme by primer design with flanking restriction sites followed by PCR amplification for cloning downstream of the GFP coding sequence in the *Pichia* expression vector pTZ-p04_GFP.

#### 2.1.5 Spike

The CoV2-Spike-D614G plasmid [a gift from Jennifer Doudna, Addgene plasmid #177960; (Syed et al., 2021)], containing Spike’s coding sequence from the Wuhan variant of SARS-Cov-2, was used for the preparation of the Spike’s cDNA by digestion with *Sac*I and *Xho*I (NEB) and creating blunt ends using Klenow. The Trastuzumab_LC_iPLUS was digested with *EcoR*V (NEB) and *Xba*I (NEB), followed by blunting with Klenow to remove trastuzumab LC and insert Spike cDNA (Spike_iPLUS). The iPLUS sequence was removed by *EcoR*V and *Xba*I digestion, followed by blunting with Klenow and re-ligation of the plasmid.

#### 2.1.6 Spike_iPLUSv2

The Spike_iPLUS plasmid was used as backbone for the preparation of the vectors containing iPLUSv2 by removal of iPLUS by digestion with *EcoR*I and *Kpn*I. For the iPLUSv2 preparation, pGEM-4 (Promega) digested with *Sac*I and *Kpn*I was used to subclone the annealead oligos containing iPLUSv2 sequence (Supplementary Table S1) flanked by digested *Sac*I and *Kpn*I restriction sites (pGEM-4-iPLUSv2). A PCR amplification of the pGEM4_iPLUSv2 was performed to obtain iPLUSv2 using pGEM4-F and pGEM4-R primers (Supplementary Table S1) and the PCR product was digested with *EcoR*I and *Kpn*I and cloned in the digested Spike_iPLUS plasmid backbone to obtain Spike_iPLUSv2.

#### 2.1.7 MAGEC2

The pDONR223_MAGEC2_WT [a gift from Jesse Boehm & William Hahn & David Root, Addgene plasmid #81862; (Kim et al., 2016)] was used as template to amplify *MAGEC2* by PCR using MAGEC2_F_XbaI and MAGEC2_R_EcoRV primers (Supplementary Table S1). The purified PCR product was digested with *Xba*I and *EcoR*V and cloned in the Trastuzumab_LC plasmid digested with the same restriction enzymes to remove *LC* and insert *MAGEC2*.

#### 2.1.8 MAGEC2_iPLUSv2

An inverse PCR was performed using primers containing the iPLUSv2 sequence (MAGEC2_inv_F and MAGEC2_inv_R; Supplementary Table S1) using MAGEC2 as DNA template. PCR products were treated with *Dpn*I (NEB) to eliminate parental DNA and ligated using T4 DNA ligase (NEB).

Sanger Sequencing was performed at the i3S Genomic Core Facility using the 3500 Genetic Analyzer sequencer (Applied Biosystems) to confirm that all vectors used in this study had the correct sequence.

### 2.2 Cell culture

HeLa cells were grown and maintained with Dulbecco’s Modified Eagle Medium (DMEM) with GlutaMAX (Gibco, Thermo Fisher Scientific), supplemented with 10% fetal bovine serum (FBS; Gibco, Thermo Fisher Scientific) and 1% penicillin-streptomycin solution (Gibco, Thermo Fisher Scientific). Cells were maintained in an incubator at 37°C, with 5% CO_2_, and were subcultured every 3–4 days.

ExpiCHO-S cell line (Thermo Fisher Scientific) was maintained in 30 mL of ExpiCHO Expression Medium (Gibco, Thermo Fisher Scientific), in 125 mL Nalgene Single-Use PETG Erlenmeyer Flasks with Plain Bottom (Thermo Fisher Scientific). Cells were kept in an incubator at 37°C, with 8% CO_2_ and 80% of relative humidity, with agitation at 125 rpm. When cells reached a concentration between 4 × 10^6^ and 6 × 10^6^ viable cells/mL, routine splitting to the desired dilution was performed, following the manufactures procedure.

### 2.3 Cell transfection

HeLa cells were transfected with the different vectors using Lipofectamine 2000 reagent (Thermo Fisher Scientific). For that, HeLa cells were seeded in 24-well plates, in a concentration that allowed them to reach 70%–90% confluency in the following day. For transfections, 1 µL of Lipofectamine 2000 and 500 ng of each plasmid was diluted in Opti-MEM (Thermo Fisher Scientific), following the manufacturer’s protocol. Cells were allowed to grow at 37°C for 48 h, after which they were collected and processed, either for total RNA or protein extraction. ExpiCHO-S cells were transfected using ExpiFectamine CHO reagent (Thermo Fisher Scientific), following the manufacturer’s protocol. In the day prior to transfection (day −1), cells were diluted to a final density of 4 × 10^6^ viable cells/mL, in order to reach a density of 7 × 10^6^–10 × 10^6^ viable cells/mL in the following day (day 0). On the day of transfection, cell viability was assessed, and cells were split to a concentration of 6 × 10^6^ viable cells/mL in a 25 mL final volume. Cells were transfected with 20 μg of plasmid DNA in a final volume of 20 μL and, for Transtuzumab constructs, a 2:1 LC:HC ratio was followed. Following the Standard Protocol for transfection, ExpiFectamine CHO Enhancer and ExpiCHO Feed (Thermo Fisher Scientific) were added the day after transfection (day 1) and cells were kept at 37°C with 8% CO_2_ for the remaining 9 days. At the time of cell harvest for downstream analysis, cell viability was at least 75%.

### 2.4 RNA extraction

Total RNA extraction was performed using TRIzol Reagent (Ambion by Life Technologies) following the manufacturer’s standard protocol, with the exception that all RNA precipitations were carried out at −80°C, during at least 2 h, and using 1 μL of glycogen (15 mg/mL; Roche). Quantification and quality assessment of RNA was performed using the Nanodrop 1000 Spectrophotometer (Thermo Fisher Scientific). RNA samples were stored at −80°C until further use.

### 2.5 DNase I treatment

To eliminate any potential contamination from genomic and/or plasmid DNA in the RNA samples, these were incubated with DNase I (Roche) for 2 h at 37°C following the manufacturer protocol.

### 2.6 cDNA synthesis

Following DNase I treatment, 1 µL of Random Hexamers (50 µM) and 1 µL of dNTPs mix (10 nM each) (Thermo Fisher Scientific) were added and samples were incubated at 65°C for 5 min followed by 5 min at 4°C. After that, 4 µL of 5x SSIV Buffer (Invitrogen), 1 µL of dithiothreitol (DTT; 100 mM; Invitrogen), 0.5 µL of Ribolock Recombinant RNase Inhibitor (40 U/µL; Thermo Fisher Scientific), and 0.5 µL of Superscript IV (SSIV) Reverse Transcriptase (200 U/µL; Invitrogen) were added to each sample. As a negative control to detect genomic DNA, the same mixtures were prepared for each sample, but without the addition of SSIV RT (-RT). Samples were incubated for 10 min at 23°C, 10 min at 55°C, and finally, 10 min at 80°C to inactivate the enzyme. Samples were stored at −20°C until further use.

### 2.7 Reverse transcription quantitative real-time PCR (RT-qPCR)

RT-qPCRs were prepared using 1 μL of cDNA and 9 μL of a mixture containing 5 μL of 2x SYBR Select Master Mix (Applied Biosystems), 0.125 μL (0.125 μM) of each primer (listed in Supplementary Table S1) and 3.75 μL of nuclease-free water. Samples were run in triplicate on a 7500 Fast Real-Time PCR System (Applied Biosystems), using the program: 50°C for 2 min, 95°C for 2 min; 40 cycles at 95°C for 15 s, 60°C for 1 min followed by a melt curve stage. Non-template controls and -RT samples were also used in each experiment. Results were analyzed using the ΔΔCt method (Normalized fold expression) using *ACTB* (for ExpiCHO-S cells) or *18S* (for HeLa cells) reference genes to normalize gene expression (Pfaffl, 2001).

### 2.8 Enzyme-linked immunosorbent assay (ELISA)

For recombinant trastuzumab protein quantification, the human IgG (total) uncoated ELISA kit with plates (Invitrogen) was used. For sample preparation, ExpiCHO-S cells were collected and centrifuged at 4,000 *g* for 30 min. The supernatant containing the recombinant protein was recovered and used for the ELISA protocol using 1:250 or 1:500 dilutions. For each condition, duplicates were made and non-transfected cells were used as negative controls. The ELISA manufacturer’s protocol was followed and the 96-well plate was read at 450 nm and 630 nm in a Synergy2 plate reader (BioTek).

### 2.9 Western blot

Whole-cell protein extracts were obtained by cell lysis with NP-40 cell lysis buffer (50 mM Tris–HCl pH 8.0, 150 mM NaCl, 1% NP-40), supplemented with 1x protease inhibitor cocktail (Sigma, P8340). Protein extracts were quantified using the Quick Bradford reagent (Bio-Rad) and 20–40 µg were added to Laemmli Buffer 2x (Bio-Rad) with 5% of β-mercaptoethanol in a 1:1 ratio, and boiled for 5 min at 95°C. Samples were run in a 3% stacking and 10% resolving Tris-glycine SDS-polyacrylamide gel and transferred into a nitrocellulose membrane, using a dry transfer method with the iBlot Gel Transfer Device (Thermo Fisher Scientific). The membrane was incubated with the respective primary antibody diluted in TBS-0.1% Tween 20 containing 5% non-fat dried milk, followed by three washes with TBS-0.1% Tween 20 and incubation with the appropriate secondary antibody. The antibodies used in this study were: anti-SARS-CoV-2 Spike mouse monoclonal antibody (1A9; GeneTex; 1:2,000 dilution), anti-α-tubulin mouse monoclonal antibody (B-5-1-2; Sigma; 1:150,000 dilution), anti-GAPDH mouse monoclonal antibody (ab8245; Abcam; 1:1,500,000 dilution), recombinant anti-MAGEC2 antibody (ab209667; Abcam; 1:2,500 dilution), goat anti-mouse IgG-HRP secondary antibody (Santa Cruz Biotechnologies; 1:10,000 dilution) and goat anti-rabbit IgG-HRP secondary antibody (Santa Cruz Biotechnologies; 1:10,000 dilution). Detection was achieved using enhanced chemiluminescence (ECL Prime, GE Healthcare) and the immunoblots were visualized on the ChemiDoc XRS + System (Bio-Rad). Quantification of the protein intensity was performed using the Image lab Software (Bio-Rad).

### 2.10 Recombinant GFP protein expression in *Pichia pastoris*

These experiments were done as a subcontracted service by Trenzyme GmbH (Konstanz, Germany). After transformation of the expression vectors pTZ_P04_GFP (control plasmid without iPLUS sequences), pTZ_P04_GFP_iPLUS 3X and pTZ_P04_GFP_iPLUSv2 into *Pichia pastoris* strain KM71H, positive integrants (stable clones) were selected by their ability to grow in presence of Zeocin. After confirmation of proper integration at the *AOX* locus by PCR based methods, five stable clones per construct were analyzed in duplicates for their ability to synthesize GFP after induction with methanol (0.5% methanol every 24 h) in a 72 h experiment. Screening process was done in a microbioreactor system (BioLector, equipped with 48-well “flower plates”) in 48 -well format, sample volume up to 1,000 µL per well in all clones in parallel.

### 2.11 Statistical analyses

Data are presented in the graphs as mean ± SD (standard deviation) and are from three independent experiments. Statistical differences were tested using two-tailed unpaired t-test. Only *p*-values <0.05 were considered statistically significant. All graphs were done using GraphPad Prism version 8.01 (GraphPad Software).

## 3 Results

### 3.1 Incorporation of iPLUS in a mammalian expression vector doubles the production of trastuzumab, a recombinant monoclonal antibody used in cancer immunotherapy

**FIGURE 1:**
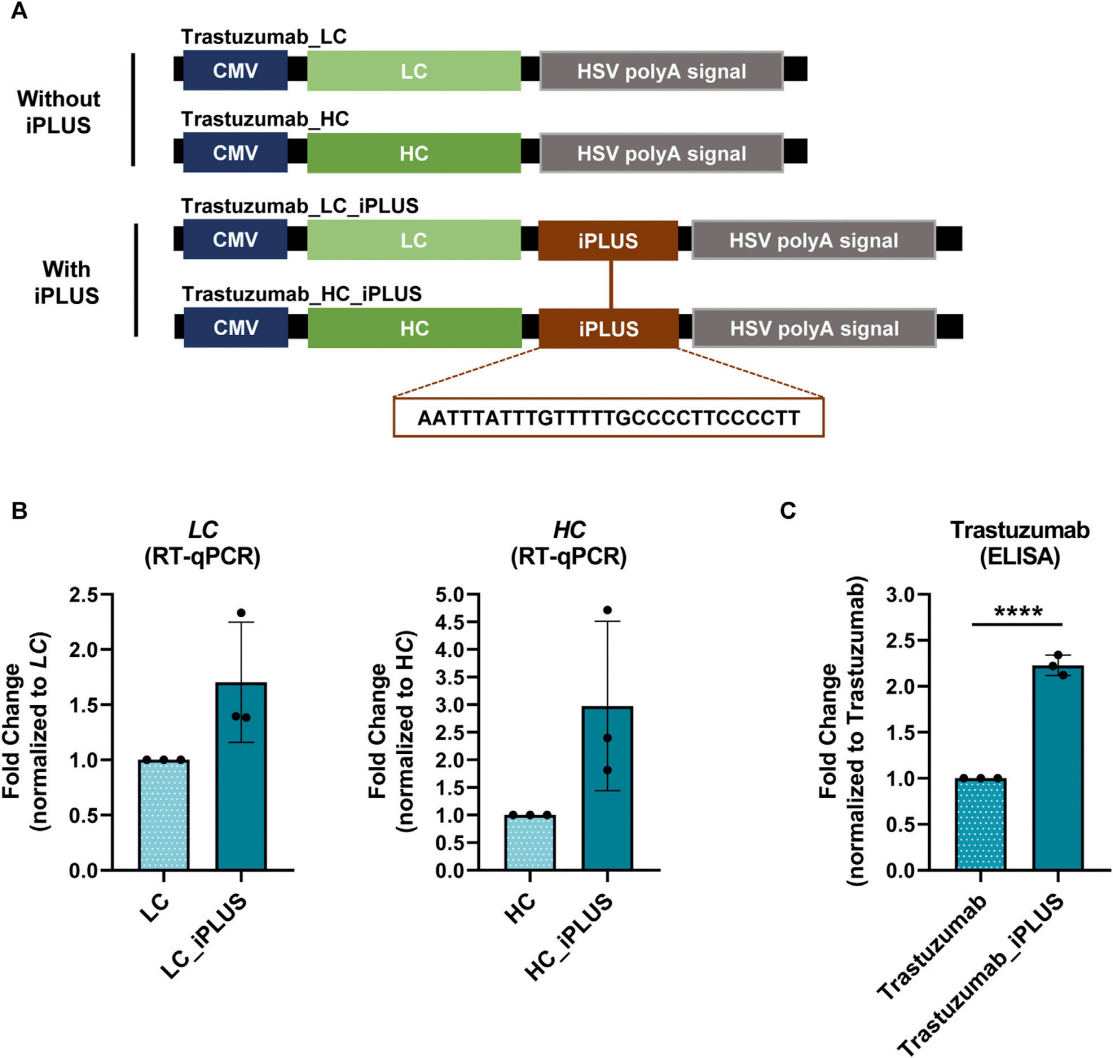
iPLUS doubles trastuzumab recombinant protein levels. **(A)** Schematic representation of the constructs containing the light chain (LC) and heavy chain (HC) of trastuzumab, as well as the constructs with the iPLUS sequence, transfected in ExpiCHO-S cells. All vectors contain a full-length cytomegalovirus (CMV) promoter and the herpes simplex virus type 1 thymidine kinase polyadenylation signal (HSV TK PAS). The 28-nucleotide iPLUS sequence is depicted in the constructs scheme. **(B)** Trastuzumab *LC* and *HC* mRNA levels were assessed by RT-qPCR and normalized to *ACTB*. Fold change to LC and HC constructs without iPLUS are represented. Data is represented as mean ± standard deviation (SD) of three independent experiments. Statistical analyses to identify differences were conducted using a two-tailed unpaired t-test. **(C)** Trastuzumab protein levels were measured by ELISA to human total IgG, and fold change trastuzumab without iPLUS is shown. Data is represented as mean ± standard deviation (SD) of three independent experiments. Statistical analyses to identify differences were conducted using a two-tailed unpaired t-test. Significant *p*-value is indicated in the graph by asterisks (****p < 0.0001).

The value of the biopharmaceutical industry has been pushed by the recombinant monoclonal antibodies (mAbs) market (Walsh, 2018). Therefore, we applied iPLUS to the production of trastuzumab, a human recombinant monoclonal antibody used in cancer immunotherapy (Albanell and Baselga, 1999; Krasniqi et al., 2019). As other mAbs, trastuzumab is composed of four polypeptide chains: two identical light chains (LC) and two identical heavy chains (HC), bound by covalent bonds, mainly disulfide bonds, forming a heterodimeric Y-shaped structure (Castelli et al., 2019). We started by subcloning trastuzumab LC and HC chains separately in the pcDNA3.4 expression vector (Figure 1A). We chose pcDNA3.4 as it is widely used in industrial settings for monoclonal antibodies production (Kosuge et al., 2020). This vector contains a full-length CMV promoter that drives high levels of expression, and was modified by the vector’s industry (Thermo Fisher Scientific) to include the WPRE downstream of the multiple cloning site, which increases the expression of several proteins, such as GFP and luciferase (Zufferey et al., 1999).

Our hypothesis was that the inclusion of the iPLUS in the pcDNA3.4 expression vector could further improve the yield of production. We thus incorporated in the LC and HC containing vectors the 28-nucleotide long iPLUS non-coding sequence downstream of WPRE and upstream of the herpes simplex virus (HSV) type 1 thymidine kinase (TK) PAS (Figure 1A). Co-transfection of LC:HC antibody chains containing vectors with and without iPLUS was performed in ExpiCHO-S cells and antibody protein levels were quantified by an IgG ELISA assay and mRNA levels evaluated by RT-qPCR. ExpiCHO-S cells were selected as they are commonly used in the biopharmaceutical industries for therapeutic recombinant protein production due to their ability to survive in animal-component-free medium and grow in suspension at high densities. As can be observed in Figure 1B, vectors containing iPLUS result in an increase in *LC* and *HC* mRNA levels, as well as in a 2.2-fold increase in trastuzumab protein levels (Figure 1C). These results show that iPLUS doubles the production of this important therapeutic monoclonal antibody at the laboratory scale.

### 3.2 iPLUS 3X and the iPLUS variant sequence iPLUSv2 highly improve recombinant protein expression levels in *Pichia pastoris*

The next step was to evaluate the use of iPLUS in yeast. *Pichia pastoris* may use methanol as a carbon source, allowing the induction of protein expression in a highly controlled manner through the AOX1 promoter, making it especially suitable for large-scale and industrial applications. In fact, *Pichia* is widely used by the biopharmaceutical industry to produce recombinant proteins (Cregg et al., 2000).

**FIGURE 2:**
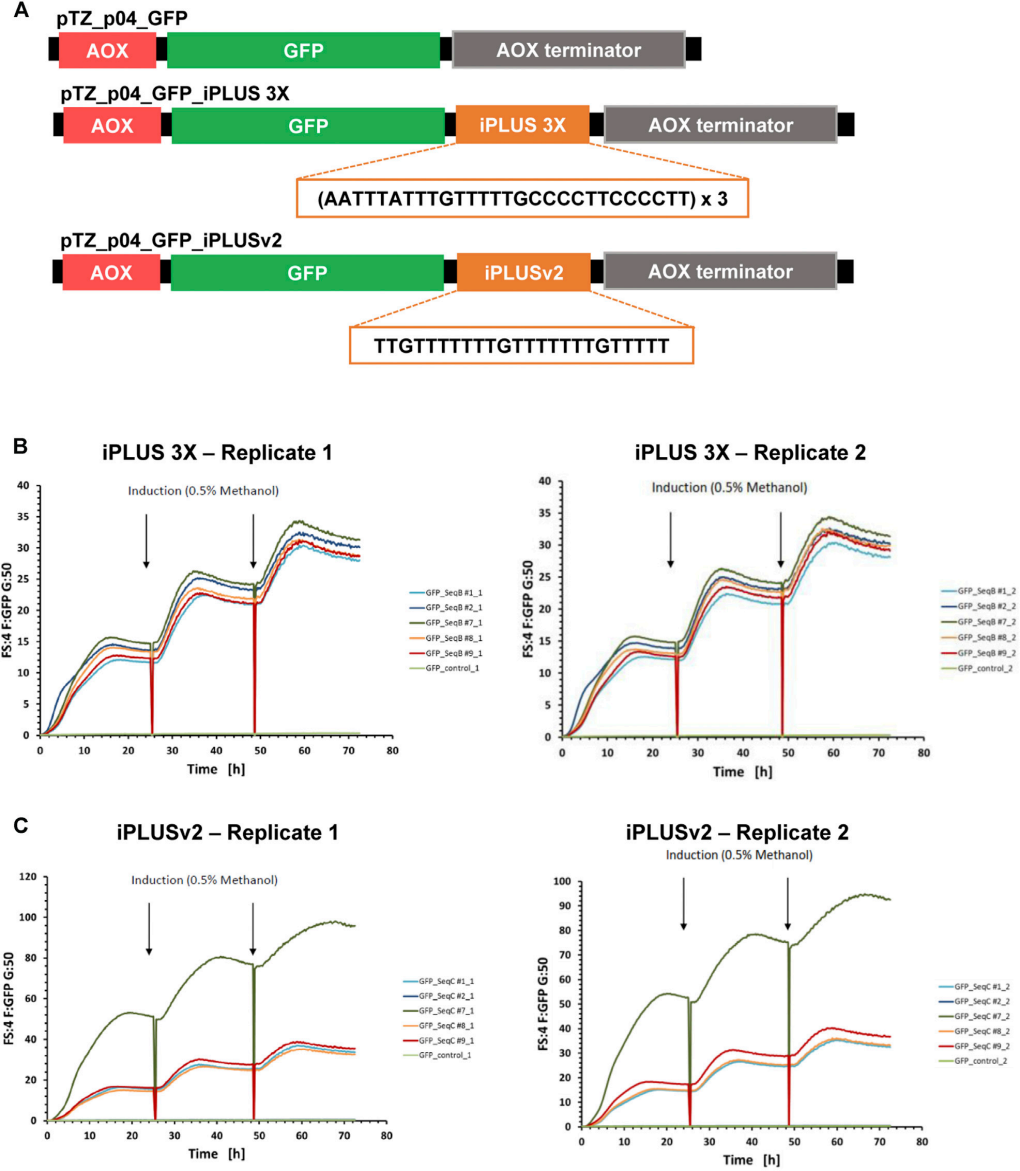
iPLUS 3X and iPLUSv2 boost GFP expression levels in *Pichia pastoris.* **(A)** Scheme of the *Pichia pastoris* pTZ_p04 expression constructs containing the GFP coding sequence, as well as the constructs with the iPLUS sequence in tandem 3X and the iPLUS variant iPLUSv2. All pTZ_p04 vectors contain a methanol inducible alcohol oxidase gene I (AOX) promoter and an AOX terminator. The 84-nucleotide iPLUS 3X sequence and the 24-nucleotide iPLUSv2 sequence introduced downstream the GFP coding sequence are depicted in the constructs. **(B)** Both iPLUS 3X and **(C)** iPLUSv2 increase GFP expression in *Pichia*. Five different *Pichia* stable clones for iPLUS 3X **(B)** and iPLUSv2 **(C)** were analysed in duplicates (replicates 1 and 2) in a microbioreactor system for their ability to synthesize GFP after induction with 0.5% methanol at 24 and 48 h time-points. iPLUS containing constructs show a strong increase in GFP expression, 114-fold for iPLUS 3X and 303-fold for iPLUSv2, in the best performing clones (clones #7), in comparison with the control clone expressing GFP (GFP-control; light green).

We used the GFP reporter protein as a read-out for the use of iPLUS in *Pichia pastoris*, and started by making GFP constructs with and without iPLUS (GFP-control) sequences by gene synthesis. The iPLUS constructs contained the iPLUS sequence in tandem 3X (iPLUS 3X), to test if the inclusion of several iPLUS repeats would increase protein levels, and a variant sequence of iPLUS (iPLUSv2), which corresponds to the most conserved region of the iPLUS contained in *Drosophila*’s *polo* gene (Oliveira et al., 2019), also repeated 3 times (Figure 2A). These sequences were then cloned into a *Pichia* expression vector having GFP under the control of methanol inducible AOX promoter (Figure 2A). After *Pichia* transformation with the different constructs, five stable clones (positive integrants) of each construct were selected and analyzed for their ability to synthesize GFP after induction with methanol. The parallel screening of all clones in a microbioreactor system demonstrates that both iPLUS 3X (Figure 2B) and iPLUSv2 (Figure 2C) can significantly increase GFP expression in *Pichia*. In comparison to the control GFP without any iPLUS sequence (Supplementary Figure S1), the best performing clones showed an increase in GFP expression of 114-fold for iPLUS 3X and 303-fold for iPLUSv2.

This proof-of-concept experiment underscore the versatility and efficacy of iPLUS and reveals that different iPLUS variants, such as the new iPLUSv2, enhance protein production in *Pichia pastoris* expression systems. Furthermore, it clearly shows that iPLUS may be a valuable asset for the improvement of *Pichia* expression vectors with biopharmaceutical applications.

### 3.3 iPLUSv2 enhances Spike’s mRNA and protein levels in different cell lines

In the post COVID-19 pandemic landscape, mRNA vaccines are positioned as a dynamic and groundbreaking technology platform for addressing global health threats (Lorentzen et al., 2022). However, moving from a novel technology to a cornerstone of vaccination strategies in a short time opens space for improvements, such as in the capacity of increasing antigen production levels.

Given our results in improving trastuzumab expression in mammalian cells, as well as with the new variant iPLUSv2 in *Pichia*, we hypothesized that the inclusion of the new activator sequence, iPLUSv2, in the 3′end of Spike’s coding sequence could be a valuable asset for the mRNA vaccine technology. In fact, the inclusion of *cis*-regulatory sequences in the 3′ end of Spike was also applied by Moderna and Pfizer in their COVID-19 mRNA vaccines (Xia, 2021).

**FIGURE 3:**
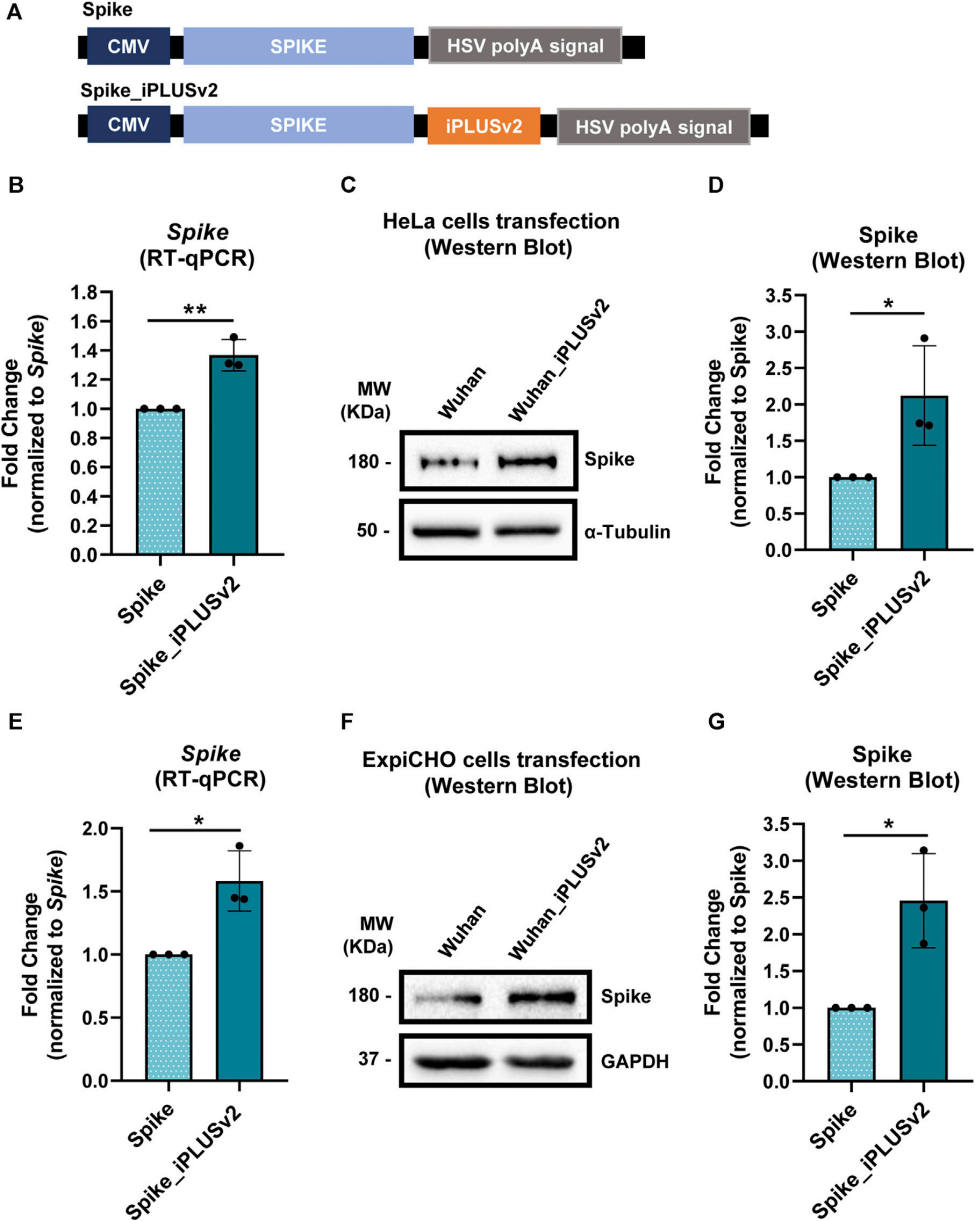
iPLUSv2 leads to an increase in Spike’s mRNA levels and protein production, in both HeLa and ExpiCHO-S cell lines. **(A)** Schematic representation of the constructs containing Spike’s cDNA and the iPLUS variant, iPLUSv2. Additionally, these vectors contain a full-length CMV promoter and the HSV TK PAS. These vectors were used for HeLa and ExpiCHO-S cells transfection, after which protein was quantified and mRNA levels were measured. **(B)** Spike’s mRNA levels were measured by RT-qPCR after HeLa cells transfection and normalized to the housekeeping *18S*. Fold change was calculated in relation to Spike. Data are shown as mean + standard deviation (SD) for three different experiments (*n* = 3). Statistical significancy was determined by two-tailed unpaired t-test. **(C)** Representative image of Spike’s protein levels evaluated by Western blot after HeLa cells transfection, being the protein quantification levels plotted for each condition, for three independent experiments (*n* = 3) in **(D)**. **(D)** Spike’s protein quantification was calculated using α-Tubulin as the loading control for normalization, followed by fold change analyses, normalized to Spike. Data are shown as mean + standard deviation (SD). Statistical significancy was determined by two-tailed unpaired t-test. **(E)** Spike’s mRNA levels were measured by RT-qPCR after ExpiCHO-S cells transfection and normalized to the housekeeping hACTB. Fold change was calculated in relation to Spike. Data are shown as mean + standard deviation (SD) for three different experiments (*n* = 3). Statistical significancy was determined by two-tailed unpaired t-test. **(F)** Representative image of Spike’s protein levels evaluated by Western blot after ExpiCHO-S cells transfection, being the protein quantification levels plotted for each condition, for three independent experiments (*n* = 3) in **(G)**. **(G)** Spike’s protein quantification was calculated using GAPDH as the loading control for normalization, followed by fold change analyses, normalized to Spike. Data are shown as mean + standard deviation (SD). Statistical significancy was determined by two-tailed unpaired t-test. Significant *p*-value is indicated in the graph by asterisks (*p < 0.05 and **p < 0.01).

As in the trastuzumab experiments, the pcDNA3.4 constructs were used (Figure 3A), with iPLUSv2 downstream of Spike. We tested two different cell lines, HeLa and ExpiCHO-S. Human HeLa cells are excellent models for proof-of-concept experiments, due to their easiness in manipulation and analyses. ExpiCHO-S cells were selected not only because they are commonly used by the industry but also as iPLUS increases trastuzumab expression in these cells (Figure 1).

As observed previously for *Pichia* (Figure 2), in HeLa cells the inclusion of iPLUSv2 leads to an increase in both mRNA and protein levels of Spike (Figures 3B–D), in comparison to the control vector without iPLUSv2. For the protein levels, this increase is approximately 2.1-fold, while for the mRNA levels the increase is approximately 1.4-fold. In ExpiCHO-S cells, iPLUSv2 promoted, once again, an increase in both Spike’s mRNA and protein levels, as illustrated in Figures 3E–G. Here, the increase in protein production is approximately 2.5-fold, while for the mRNA levels the increase is approximately 1.6-fold.

These results clearly show that iPLUSv2 can increase Spike’s mRNA levels and double protein production, in two different cell lines from different species (human and hamster).

### 3.4 MAGEC2 mRNA and protein levels increase with iPLUSv2

The mRNA vaccines are a potential immunotherapy solution to combat various types of cancer, prompting the immune system to destroy cancer cells by triggering a specific and enduring immune response against tumor antigens (TAs), inhibiting tumor growth (Miao et al., 2021). Nevertheless, in clinical trials it has been observed that although there is a certain level of positive response from patients (Papachristofilou et al., 2019), it does not seem to be a sufficiently robust response to cause tumor recession or an increase in overall survival for all the patients (Chehelgerdi and Chehelgerdi, 2023), leading to termination of their production. For that reason, pharmaceutical companies ought to incorporate novel molecular tools to improve cancer mRNA vaccines, to promote good immunological responses.

Therefore, our next step was to assess whether subcloning iPLUSv2 downstream of the melanoma antigen family C2 (MAGEC2) would result in increased mRNA levels and consequent protein production. For these proof-of-concept experiments, MAGEC2, a tumor-specific antigen for non-small cell lung cancer (NSCLC), was selected since it is included in CureVac’s CV9202 NSCLC mRNA vaccine and whose information was made public (Papachristofilou et al., 2019). CV9202 is a vaccine encoding 6 tumor associated antigens, being MAGE-C2 one of them. Antigen-specific immune responses were detected in a phase Ib study enrolling 26 patients with stage IV NSCL (Papachristofilou et al., 2019; Sebastian et al., 2019).

**FIGURE 4:**
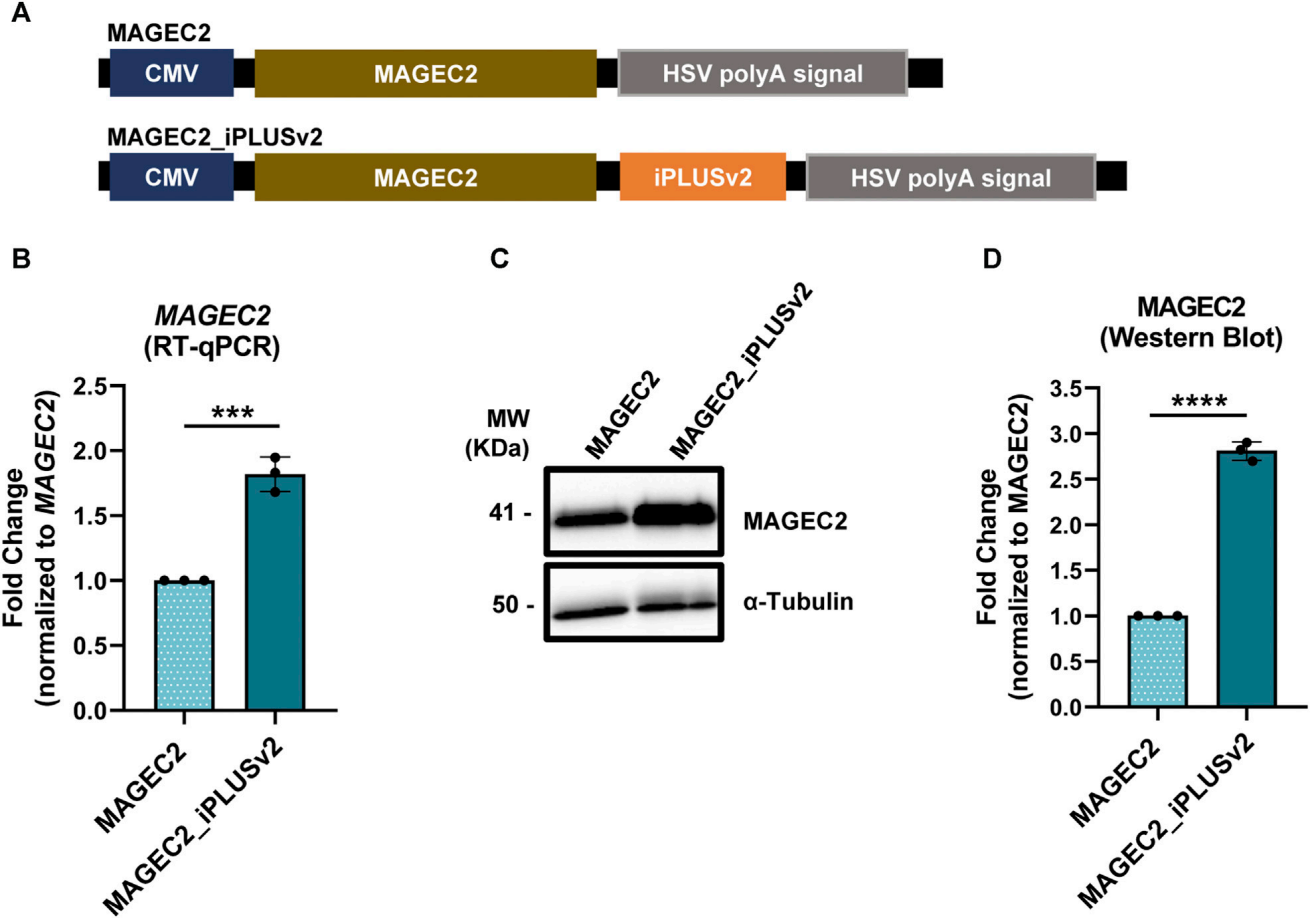
iPLUSv2 increases MAGEC2 mRNA and protein levels. **(A)** Schematic representation of the constructs, containing the *MAGEC2* coding sequence and the iPLUS variant, iPLUSv2, transfected in HeLa cells. Vectors contain a full-length CMV promoter and the HSV TK PAS. **(B)** *MAGEC2* mRNA levels increase in the presence of iPLUSv2. mRNA levels were measured by RT-qPCR and normalized to *18S*. Fold change to MAGEC2 without iPLUSv2 is represented as mean ± standard deviation (SD) of three independent experiments. Statistical analyses to identify differences were done using a two-tailed unpaired t-test. Significant *p*-value is indicated in the graph by asterisks (***p < 0.001). **(C,D)** MAGEC2 protein levels increase >2-fold in the presence of iPLUSv2. **(C)** Representative image of MAGEC2 protein levels evaluated by Western blot, with α-Tubulin as a loading control. **(D)** Quantification of MAGEC2 protein levels was calculated using the respective loading control for normalization, followed by fold change analyses to MAGEC2 without iPLUSv2. Data is represented as mean ± standard deviation (SD) of three independent experiments. Statistical analyses to identify differences were conducted using a two-tailed unpaired t-test. Significant *p*-value is indicated in the graph by asterisks (****p < 0.0001).

As before, the initial step was the construction of vectors containing the *MAGEC2* with and without iPLUSv2 downstream of this antigen (Figure 4A) and these vectors were used to transfect HeLa cells. As it can be observed in Figures 4B, C, the inclusion of iPLUSv2 sequence in *MAGEC2* vectors resulted in an increase in approximately 1.8-fold in *MAGEC2* mRNA levels (Figure 4B) and a 2.8-fold increase in MAGEC2 protein levels, in comparison to vectors without this sequence (Figures 4C, D).

As observed with iPLUSv2 in Spike or iPLUS in trastuzumab, these results show that the presence of iPLUS non-coding sequences in vectors used for recombinant protein expression can at least double protein production. Moreover, the impact of iPLUSv2 in MAGEC2 bears substantial implications for its application in cancer mRNA vaccines, as the increase in antigen production certainly influences the efficiency of the process.

## 4 Discussion

*Cis*-regulatory sequences present in the 3′UTR that impact both mRNA and protein production, should be further investigated to be applied to enhance the production of biopharmaceuticals (O,Flaherty et al., 2020; Kirshina et al., 2023).

The existence of U-rich USEs as auxiliary regulatory elements has been widely recognized since they were originally described in viral mRNAs (DeZazzo and Imperiale, 1989; Russnak and Ganem, 1990; Darmon and Lutz, 2012). In the Human Immunodeficiency Virus 1 (HIV-1), for example, different termination sites exist and it is a USE in the vicinity of the 3′ long terminal repeats (LTR) PAS that aids in the production of the correct length transcript, disregarding the PAS present in the 5′LTR (Valsamakis et al., 1991). It was initially thought that these sequences were specific to viral genomes, but this notion was questioned when different examples in cellular genes were discovered, such as in complement C2, Lamin B2, cyclooxygenase-2 or prothrombin, in which USEs mode of action has been described (Moreira et al., 1995; Brackenridge et al., 1997; Hall-Pogar et al., 2005; Danckwardt et al., 2011). For instance, we have showed that *polo*’s USE in *Drosophila* is essential for *polo* distal PAS selection and has a function in increasing *polo* mRNA and protein levels (Pinto et al., 2011; Oliveira et al., 2019). Moreover, microinjection of GFP-USE in one-cell stage zebrafish embryos increases GFP levels demonstrating a conservation of the role of the USE in increasing protein levels *in vivo* (Eufrásio et al., 2023). We then tested if the USE could function downstream of a reporter gene in a mammalian cell model, thus showing that the USE is also able to increase luciferase activity in HeLa cells (Oliveira et al., 2020). The results obtained with *polo*´s USE *in vivo* in two animal models and *in vitro* in Hela cells prompt us to patent the use of this sequence in several applications including in the production of recombinant proteins with therapeutic use and mRNA vaccines and re-named it as iPLUS (Oliveira et al., 2020). We have previously described the mechanism of action of USEiPLUS and showed that iPLUS regulates several steps of gene expression. By liquid chromatography-tandem mass spectrometry (LC-MS/MS) analysis we demonstrated that PTBP1 binds to iPLUS *RNA* and using PTBP1 mutants we showed that it increases mRNA 3′ end formation, and consequently, mRNA levels (Oliveira et al., 2019). Additionally, we showed by UV crosslinking and immunoprecipitation assays that HuR (Brennan and Steitz, 2001; Hinman and Lou, 2008; Ho et al., 2020), a RBP with a well-defined role in mRNA stability and translation efficiency, also binds to *in vitro* transcribed iPLUS *RNA*, thus increasing protein levels (Eufrásio et al., 2023). Despite the fact that USE’s/iPLUS physiological roles and mechanistic mode of function have been thoroughly investigated, this is the first time that a USE/iPLUS has been employed with the purpose of boosting protein production in different expression systems.

Here, we have expanded upon our earlier findings by exploring the potential of the non-coding iPLUS sequence to enhance the production of relevant biopharmaceuticals, including monoclonal antibodies used in immunotherapy and antigens employed in mRNA vaccines. Our results show the versatility of iPLUS by demonstrating its efficacy in improving the production of relevant recombinant proteins across various models. We have showed that in a recombinant monoclonal antibody commonly used in cancer immunotherapy, trastuzumab, iPLUS increases *LC* and *HC* mRNA levels and doubles trastuzumab protein levels in ExpiCHO-S cells. Next, we showed that in *Pichia,* a strain of yeast extensively used in recombinant protein production, the inclusion of iPLUS and a new iPLUS variant sequence in *Pichia* expression vectors containing GFP strongly impacts GFP expression levels, in at least 100-fold. Upon these consistent results, we turned our attention to a different kind of biopharmaceutical, in high demand at the moment, mRNA vaccines. For Spike, the antigen used in the COVID-19 mRNA vaccines, we observed again an increase in both mRNA and protein levels, in two different models, HeLa and ExpiCHO-S cells. The same effect was observed when we evaluated a different antigen tested in a lung cancer mRNA vaccine, MAGEC2. These results clearly show that in the three tested models (HeLa, ExpiCHO-S and *Pichia*) and with various proteins, iPLUS or its variant sequence iPLUSv2, consistently exhibit a beneficial impact in increasing both mRNA and protein levels.

These results produce proof-of-concept data of the applicability of the use of iPLUS in the biopharmaceutical industry and pave the way for further scale up experiments, in an industrial context. In yeast, there is evidence that the regulatory role of sequences present in the 3′UTR is conserved (Shalgi et al., 2005; Ito et al., 2020; Savinov et al., 2021), but this is still a rather unexplored field of research. When referring specifically to *Pichia pastoris*, the knowledge is even more limited, so there is still room for improvements in this area, as we have demonstrated here by applying iPLUS to this expression system. This also opens the possibility for applying this technology to other recombinant DNA technologies, to increase the production of antibodies for immunotherapy, different mRNA vaccines antigens and possibly other therapeutic proteins, such as hormones or cytokines. Furthermore, it is possible that iPLUS presents a synergistic effect with existing non-coding regulatory sequences located at the 3′ end of expression systems, such as the WPRE, suggesting new applications for iPLUS and offering an opportunity to further enhance commercially available vectors.

Indeed, both iPLUS and iPLUSv2 offer numerous valuable advantages for their inclusion in vectors used in an industrial environment. As non-coding sequences, they avoid interference with the coding region of the protein or antigen of interest, avoiding additional regulatory steps. Their short size (28 or 25 nucleotides) facilitates easy subcloning into various expression systems or vectors without requiring complex laboratory procedures. Furthermore, iPLUS proves to be a versatile asset, as it functions across different expression systems, proteins, and mRNAs thus enabling the selection of a system tailored to the unique characteristics of each mRNA and protein (Oliveira et al., 2020).

In summary, iPLUS underscores the importance of the *cis*-acting elements present in the 3′UTR in the regulation of gene expression, highlighting its potential application for the development of different vectors and biologics. Our findings clearly show that iPLUS incorporation into vectors used in different models has the potential to be used by the biopharmaceutical industry to increase the yield of protein production, thereby reducing production costs.

## Data Availability

The raw data supporting the conclusion of this article will be made available by the authors, without undue reservation.
