# Supplementary material for: Application of the iPLUS non-coding sequence in improving biopharmaceuticals production
Source: Front Bioeng Biotechnol. 2024 Feb 6;12:1355957. doi: 10.3389/fbioe.2024.1355957 (PMC10876878; doi:10.3389/fbioe.2024.1355957)
Supplement: Supplementary file 1 [file DataSheet2.PDF]

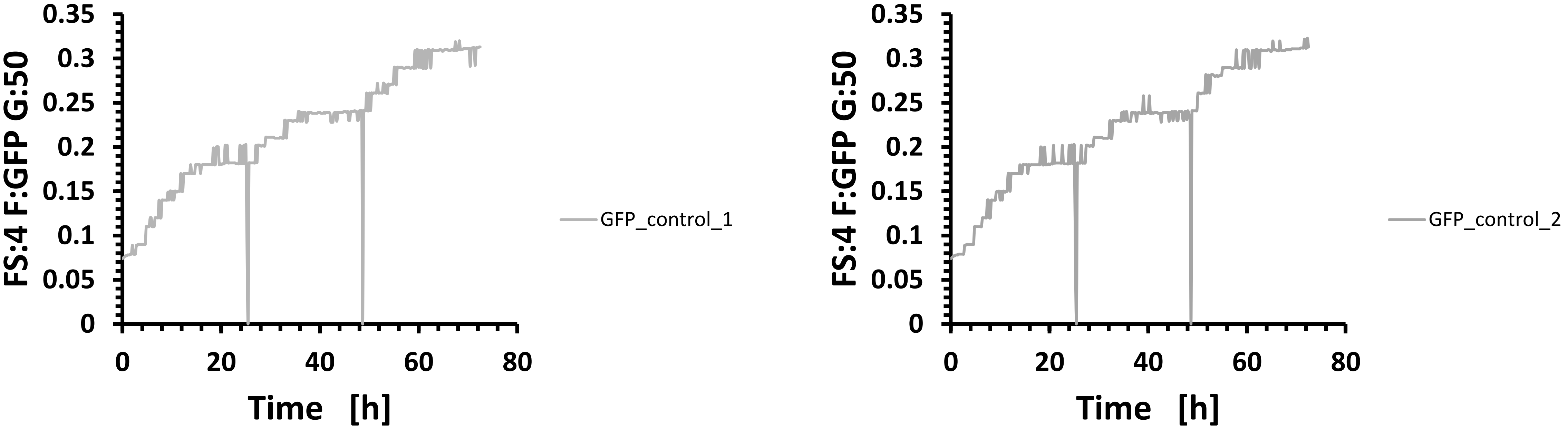

Supplementary Figure 1 - Close-up views of the curves of the control alone show significant response upon methanol-mediated induction (replicates 1 and 2) in a microbioreactor system for their ability to synthesize GFP after induction with 0.5% methanol at 24 h and 48 h time-points.
