# Supplementary material for: Application of the iPLUS non-coding sequence in improving biopharmaceuticals production
Source: Front Bioeng Biotechnol. 2024 Feb 6;12:1355957. doi: 10.3389/fbioe.2024.1355957 (PMC10876878; doi:10.3389/fbioe.2024.1355957)
Supplement: Supplementary file 2 [file DataSheet1.PDF]

Supplementary Table 1. Oligonucleotides used in this work.

Primer name	Sequence (5'- 3')	Application
LC+XbaI F	GATTCTAGAATGTTGCCATCACAACTCATTGGG	Trastuzumab_LC Cloning
LC-R	CTAACACTCTCCCCTGTTGAAGCT	
HC+XbaI F	GATTCTAGAATGGACTGGACCTGGAGGATCCTC	Trastuzumab_HC Cloning
HC-R	TCATTTACCCGGAGACAGGGAGAG	
iPLUS_sense	CAATTTATTTGTTTTTGCCCCTTCCCCTTGGTAC	iPLUS annealing oligos
iPLUS_antisense	CAAGGGGAAGGGGCAAAAACAAATAAATTGAGCT	
WPRE+BamHI-F	GATGGATCCACGGAAGGAGACAATACCGGAAGG	Trastuzumab_LC_iPLUS Cloning
WPRE+EcoRI-R	GTCGAATTCGTTTCAGTTAGCCTCCCCCGTTT	
iPLUSv2_sense	CTTGTTTTTTTGTTTTTTTGTTTTTGGTAC	iPLUSv2 annealing oligos
iPLUSv2_antisense	CAAAAACAAAAAAACAAAAAAACAAGAGCT	
pGEM4-F	AGGTGACACTATAGAATACACG	Amplification of the iPLUS_v2 sequence from pGEM4_iPLUSv2
pGEM4-R	CTGCAGGTCGACTCTAGA	
MAGEC2_F_XbaI	ATCGTCTAGAATGCCTCCCGTTCCAGGC	MAGEC2 Cloning
MAGEC2_R_EcoRV	CATGGATATCTCACTCAGAAAAGGAGAC	
MAGEC2_inv_F	TTTTTTGTTTTTCACGGAAGGAGACAATACCGGAAGGAAC	MAGEC2_iPLUSv2 Cloning
MAGEC2_inv_R	ACAAAAAAACAATTTCAGTTAGCCTCCCCCGTTTAAACCC	
HC_CDS_F	CCTGGTGGTTCTCTTCGTCT	RT-qPCR
HC_CDS_R	CGAGCAACCCATTCAAGACC	
LC_CDS_F	TCTGCTTCTGTTGGTGATCGT	
LC_CDS_R	AGGAGCTTTACCAGGTTTTTGTTG	
Spike_F	ATGACTCGAGTCTAGAACAAAA	
Spike_R	AGC TCG AGT CTA GAA CAA	
MAGE_F	GGTGCCCTCTGGTGTGATAC	
MAGE_R	AGAGGACTCTGGGAAGGACC	
ACTB_F	GGATGCAGAAGGAGATCACTG	
ACTB_R	CGATCCACACGGAGTACTTG	
18S_F	GCAGAATCCACGCCAGTACAAGA	
18S_R	CCCTCTATGGGCCCGAATCTT	
